# Tdrd12 Is Essential for Germ Cell Development and Maintenance in Zebrafish

**DOI:** 10.3390/ijms18061127

**Published:** 2017-06-07

**Authors:** Xiangyan Dai, Yuqin Shu, Qiyong Lou, Qiang Tian, Gang Zhai, Jia Song, Suxiang Lu, Hong Yu, Jiangyan He, Zhan Yin

**Affiliations:** 1 Department of Medical Cell Biology and Genetics, College of Preclinical Medicine, Southwest Medical University, Luzhou 646000, China; dai_xiangyan@126.com (X.D.); tian0703@163.com (Q.T.); yh829@126.com (H.Y.); 2 State Key Laboratory of Freshwater Ecology and Biotechnology, Institute of Hydrobiology, Chinese Academy of Sciences, Wuhan 430072, China; shuyuqin008@163.com (Y.S.); louqiyong@ihb.ac.cn (Q.L.); zhaigang@ihb.ac.cn (G.Z.); marksong92@126.com (J.S.); lu1000001@gmail.com (S.L.); jyhe@ihb.ac.cn (J.H.); 3 University of Chinese Academy of Sciences, Beijing 100049, China

**Keywords:** *tdrd12*, knockout, germ cell, differentiation, maintenance, zebrafish

## Abstract

The regularity of Piwi-interacting RNA (piRNA) biogenesis is crucial to germline development. Functioning as Piwi-interacting proteins, Tudor domain-related proteins (Tdrds) have been demonstrated to be involved in spermatogenesis and the piRNA pathway. In this study, zebrafish *tdrd12* was identified, and the maternal and germ cell-specific expression patterns of zebrafish *tdrd12* were observed. Utilizing TALEN (transcription activator-like effector nuclease) techniques, two independent *tdrd12* mutant zebrafish lines were generated. Although no defects were found during the generation of the primordial germ cells (PGCs) in the *tdrd12*-null fish progenies obtained from the heterozygous *tdrd12* mutant parents, all Tdrd12-deficient fish developed into infertile males. The reduced numbers and eventually loss of the germ cells by 35 days post fertilization (dpf) led to masculinization and infertility of the Tdrd12-deficient fish. Meiosis defects of the germ cells in the *tdrd12* mutants during the gonad-transitioning period were observed, revealing the indispensable functions of Tdrd12 in gametogenesis. Our studies demonstrated that zebrafish Tdrd12 is essential for germ cell development and maintenance.

## 1. Introduction

In many animals, sex determination is genetic, often accompanied by various sex chromosome systems. However, In zebrafish, it was generally accepted that the chromosomal sex determination (CSD) system seems to have been lost in domesticated or laboratory strains and developed a polygenic sex determination (PSD) system, so wild-type and domesticated representatives of the same species have different SD systems [1,2,3]. In laboratory strains, the early gonads initially develop into ovary-like juvenile gonads called “bipotential juvenile ovaries”. These “bipotential juvenile ovaries” can develop into mature ovaries in females or into testes in males following oocyte apoptosis. The increased apoptosis of oocytes in presumptive juvenile males during the sex transitioning period indicates the involvement of oocyte apoptosis in maintaining testicular and ovarian morphology in zebrafish [4]. Studies on the transcriptional manipulation of *brca2*, *fancl*, *fst-1*, and *ftz-1* have revealed the contribution of some genes in the sex determination pathway of zebrafish [5,6,7]. Currently, little is known about the precise trigger for sex determination during juvenile hermaphroditism of zebrafish.

Germ cells are progenitor cells that can transmit genetic information to the next generation [8]. The interaction between somatic and germ cells is important for gonad development in zebrafish. Germ cell-specific genes, such as *vasa* and *dnd*, play critical roles in the migration, specification, maintenance, and survival of germ cells [9,10]. The complete depletion of functional Dnd (Dead end) at the embryonic stages in zebrafish causes the complete loss of primordial germ cells as well as promotes the development of all the morphants into sterile males [10]. A certain number of germ cells in the sex transitioning periods and mature stages are also required to maintain a stable female sexual phenotype in adults [11,12,13,14]. On the other hand, in mammals such as mice, the gonad fate is induced through somatic cell contact with germ cells. However, little is known about the regulatory mechanisms of germ cells during sexual development.

Piwi proteins play a central role in germline development and gametogenesis and are required for piRNA biogenesis and function. In zebrafish, germ cells possess two Piwi proteins, Ziwi and Zili, which are proposed to conduct the biogenesis of piRNAs and destroy active transposon element mRNAs in the zebrafish germline via a feed-forward amplification cycle. Both are required for normal germ cell development [15,16]. The PIWI-piRNA pathway has been revealed to be essential for proper germ cell development and reproduction in mice, *Drosophila*, and zebrafish [17,18,19]. Among PIWI-interacting proteins, Tdrd family proteins have been revealed as important molecules for piRNA biogenesis. Twelve members of TDRD family proteins have been identified in mice since the *Drosophila* Tudor protein was discovered [20,21]. They are mainly involved in germ cell development [19]. For example, the disruption or depletion of *tdrd1*, *tdrd2*, *tdrd7*, and *tdrd9* leads to sterility in male mice mainly because of defects during spermatogenesis [21,22,23,24,25].

A few functional studies have been reported on the members of the zebrafish Tdrd family proteins. It was demonstrated that zebrafish Tdrd1 associates with piRNA targets, interacting with both Ziwi and Zili in zebrafish. Loss of Tdrd1 leads to defective nuage structures in germ cells, transposon desilencing, and the loss of germ cells in zebrafish. These observations also demonstrated the similar functions shared between zebrafish Tdrd1 and its mouse counterpart [21,26,27]. Tdrd6 is required for normal primordial germ cell formation and the accumulation of maternally inherited piRNAs in zebrafish. However, *tdrd6* mutants have normal germ cell development in adults [28]. Tdrd9 is also required for germ cell maintenance and affects piRNA accumulation, resulting in severe germ cell developmental defects in mutant zebrafish [28].

The Tdrd12 ortholog in *Drosophila* is known to interact with the essential piRNA pathway and regulates piRNA biogenesis in ovarian germ line cells [18]. TDRD12 in mice was also identified as a component of the PIWI protein MIWI2. All TDRD12-deficient mice are viable, and females are fertile. TDRD12 deficiency induces male testes atrophy resulting from the loss of MIWI2-bound piRNA, which is important for secondary piRNA biogenesis and spermatogenesis [29].

Zebrafish Tdrd12 has been deposited previously as a predicted Tdrd family protein in the NCBI database containing two Tudor domains and a DEAD (Asp-Glu-Ala-Asp) box without any functional study reports. In this study, a complete and precise zebrafish *tdrd12* mRNA sequence was identified. The phylogenetic analyses of the predicted amino acid sequence of this zebrafish Tdrd12 with the other Tdrd12 reveal highly evolutionary and phylogenetic relationships among species. A germ cell-specific expression pattern of zebrafish *tdrd12* was confirmed subsequently. Two independent Tdrd12-deficient fish lines have been generated using the TALEN (transcription activator-like effector nuclease) technique. Although no defects of the generation and migration of the PGCs were observed, formation of the juvenile ovary-like bipotential gonads in Tdrd12-deficient fish derived from the heterozygous mutant parents were observed during the early stage by 18 dpf. All Tdrd12-deficient mutants develop as infertile males exclusively. This indicates the requirement of Tdrd12 for germ cell development and maintenance at the zebrafish juvenile stage. Because maternal Tdrd12 could be inherited from heterozygous parents, as well as the infertility of the homozygous Tdrd12-deficient adults, we have no good indications at present on the roles of maternally provided Tdrd12 at early embryonic stages. Our data indicate that failure to support germ-cell development in Tdrd12-deficient fish is due to the meiosis defects that progress beyond the pachytene stage and the loss of germ-line stem cells eventually, both of which cause impaired testes without any germ cells. Thus, zebrafish Tdrd12 appears to be required for the development and maintenance of germ cells at least. We undertook a comprehensive analysis of the function of Tdrd12 in zebrafish, the results of which may shed light on its critical role in the piRNA pathway during gametogenesis.

## 2. Results

### 2.1. Cloning of Tdrd12 and Phylogenetic Analysis of the Tdrd12 Protein across Species

**Figure 1:**
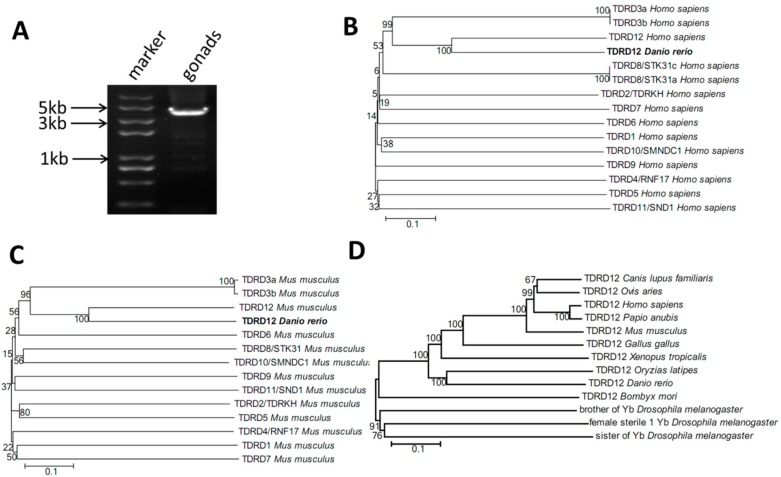
Zebrafish Tdrd12 is conserved across species. (**A**) The full-length coding region of the putative *tdrd12* mRNA was amplified from 120-dpf wild-type zebrafish gonadal cDNA samples. The appearance of the amplified fragment showed a PCR product at 4.1 k base pairs in length; (**B**) Phylogeny analysis of the putative zebrafish Tdrd12 protein and human TDRD family protein members. The results indicate that the putative zebrafish Tdrd12 is most homologous to human TDRD12. The TDRD proteins of humans were as follows: TDRD1: NP_942090.1; TDRD2/TDRKH (Tudor and KH domain-containing protein): NP_001077432.1; TDRD3a and TDRD3b: NP_001139542.1 and NP_001139543.1; TDRD4/RNF17 (RING finger protein 17): NP_001171922.1; TDRD5: NP_001186014.1; TDRD6: NP_001010870.1; TDRD7: NP_001289813.1; TDRD8c and TDRD8a/STK31 (serine/threonine-protein kinase 31): NP_001247434.1 and NP_113602.2; TDRD9: NP_694591.2; TDRD10/SMNDC1 (survival of motor neuron-related-splicing factor 30): NP_005862.1; TDRD11/SND1 (staphylococcal nuclease domain-containing protein 1): NP_055205.2; TDRD12: XP_011525773.1; (**C**) Phylogeny analysis of the putative zebrafish Tdrd12 protein and mouse TDRD family protein members. The results indicate that the putative zebrafish Tdrd12 is most homologous to mouse TDRD12. The TDRD proteins of mice were as follows: TDRD1: NP_001002238.1; TDRD2/TDRKH (Tudor and KH domain-containing protein): XP_006502201.1; TDRD3a and TDRD3b: NP_766193.3 and NP_001240684.1; TDRD4/RNF17 (RING finger protein 17): NP_001028215.1; TDRD5: NP_001128213.1; TDRD6: NP_001154838.1; TDRD7: NP_001277404.1; TDRD8/STK31 (serine/threonine-protein kinase 31): NP_084192.2; TDRD9: NP_083332.1; TDRD10/SMNDC1 (survival of motor neuron-related-splicing factor 30): NP_766017.1; TDRD11/SND1 (staphylococcal nuclease domain-containing protein 1): NP_062750.2; TDRD12: XP_017167806.1; and (**D**) Phylogenetic tree of Tdrd12 proteins in zebrafish, medaka, fly, Bombyx, frog, chicken, mouse, olive baboon, human, dog, and sheep. The proteins are as follows. *Drosophila melanogaster* (Brother of Yb): NP_649430.1 *Drosophila melanogaster* (sister of Yb): NP_001245959; *Drosophila melanogaster* (female sterile 1 Yb): NP_477494.2; *Bombyx mori*: NP_001037005.1 b; *Xenopus tropicalis*: XP_012816810.1; *Oryzias latipes*: XP_011471120.1; *Danio rerio*: this manuscript; *Gallus gallus*: XP_015147962.1; *Papio anubis*: XP_009192354.1; *Homo sapiens*: XP_011525773.1; *Ovis aries*: XP_012045510.1; *Canis lupus familiaris*: XP_013976340.1; *Mus musculus*: XP_017167806.1.

Previously, there were two sources of sequence information for putative zebrafish *tdrd12* gene sequences deposited in the NCBI and Ensembl databases (predicted Tdrd12-like, accession number: XP_017209647.1, encoding for a putative 1122 amino acid protein; and a 5’-imcomplete *tdrd12* coding region, ENSDARG00000075217, encoding for a putative 1111AA). Based on previous information in the NCBI database and our RNA-sequence data for zebrafish testis samples, we successfully obtained a complete transcript with the 5’-untranslation region (UTR), full-length coding region, and 3’-UTR, with a putative 1362 amino acid protein. The complete transcript of the coding region was amplified from wild-type zebrafish testis samples (120-dpf) with RT-PCR using designed primers (Table 1, Figure 1A) and then was confirmed by sequencing. The information of this identified zebrafish *tdrd12* has been submitted to NCBI GenBank with the accession No. KY436158.

It was reported that 12 members of the TDRD protein family were characterized in the mouse since the *Drosophila* Tudor protein was discovered [30]. Previous studies of TDRD12 in the fly, Bombyx, and mouse indicated that the functions and mechanisms were conserved [15,26]. To examine the conserved relationship between our newly identified zebrafish Tdrd12 and the other known TDRD proteins, phylogenetic trees of zebrafish Tdrd12 with the 12 known TDRD proteins from humans and mice, as well as the Tdrd12 proteins from representative fish, amphibians, poultry, and mammals, were generated using the neighbor-joining (NJ) phylogenetic method. Compared with all the known TDRD proteins from humans and mice, our newly identified putative Tdrd12 protein was most homologous to the TDRD12 proteins in mice and humans (Figure 1B,C). In addition, the NJ phylogenetic tree revealed that the zebrafish Tdrd12 protein is most homologous to the Tdrd12 of medaka, followed by that of frogs and mice (Figure 1D). To compare the Tdrd proteins identified in GenBank, we also characterized all the known Tdrd family proteins in zebrafish and annotated all additional domains of 12 members; these members all contain the conserved Tudor-domain and corresponded to the mouse Tudor family proteins. Previously, little was known about the functions and mechanisms regarding the Tdrd members in zebrafish; however, the functions of zebrafish Tdrd1, Tdrd6, Tdrd7, and Tdrd9 were mostly related to germline development (Table 2).

### 2.2. Tissue-Specific and Maternal Expression Pattern of Zebrafish tdrd12

**Figure 2:**
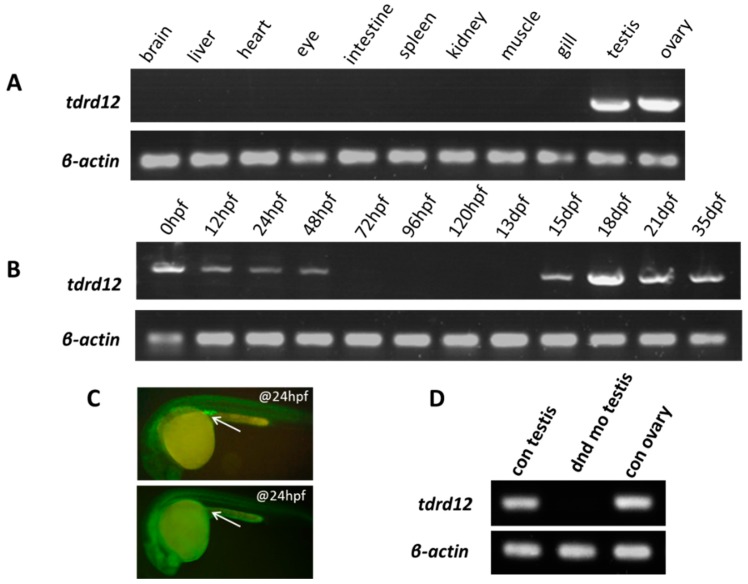
Maternal and germ cell-related expression patterns of *tdrd12*. (**A**) Tissue distribution of endogenous *tdrd12* transcripts with RT-PCR indicates its gonad-specific patterns in adult zebrafish; (**B**) The presence of endogenous *tdrd12* transcripts at different developmental stages reveals its maternal expression pattern in unfertilized eggs. The expression of zebrafish *tdrd12* is decreased in the early embryonic stage until the 48 hpf stage. The *tdrd12* transcripts reappear from 15 dpf to the adult stage in the gonads; (**C**) Presence of PGCs visualized with injected EGFP-*dnd* 3’UTR mRNA in control larva (**upper panel**, injected with control morpholino) and PGC-depleted larva (**lower panel**, injected with *dnd* morpholino) at the 24-hpf stage. The arrowheads here show the presence of zebrafish PGCs in larva; and (**D**) Presence of *tdrd12* transcripts detected in adult gonadal tissues of the control fish and PGC-depleted *dnd* morphants (no ovary). The transcripts of *β-actin2* were amplified from the same templates as an internal control to check the quality of the cDNA.

Most *tdrd* family genes are expressed predominantly in gonad tissues [30]. To examine our newly identified endogenous *tdrd12* in various tissues of adult zebrafish (120 dpf), we performed RT-PCR assays. Like most of the other *tdrd* genes reported previously, zebrafish *tdrd12* was only expressed in the gonads, in both the testis and ovary (Figure 2A). When we examined the *tdrd12* transcripts during the early embryonic stage, it was observed that *tdrd12* mRNA was maternally provided and gradually disappeared until 48 hpf; little or no mRNA was detected in the developmental stages between 3 and 13 dpf. Subsequently, the presence of *tdrd12* transcription resumed from 15 dpf and remained high until 35 dpf (Figure 2B).

Knock down of *dead end* (*dnd*), a gene important for the survival of zebrafish PGC, would ablate the PGC in zebrafish to achieve germ cell-free adult males [32]. We have generated germ cell-free adult zebrafish with specific morpholino (MO) against *dead end* (*dnd*) injected into wild-type zebrafish at the 1–2 cell stage (Figure 2C). The absence of *tdrd12* transcripts in the germ cell-free testes from the *dnd*-morphants indicates the germ cell-specific or germ cell-related expression pattern of *tdrd12* in vivo (Figure 2D). In situ hybridization assays of both embryonic stage and adult gonads reveal the maternally provided mRNA and germ cell-specific expression pattern (Figure S1A–F). However, the transcription levels of *tdrd12* might be too low to be detected using in situ hybridization assays at 11 hpf and 24 hpf (Figure S1C,D), although it could be detected by reverse transcription PCR (Figure 2B).

### 2.3. Targeted Disruption of tdrd12 Mutant Lines by TALEN

**Figure 3:**
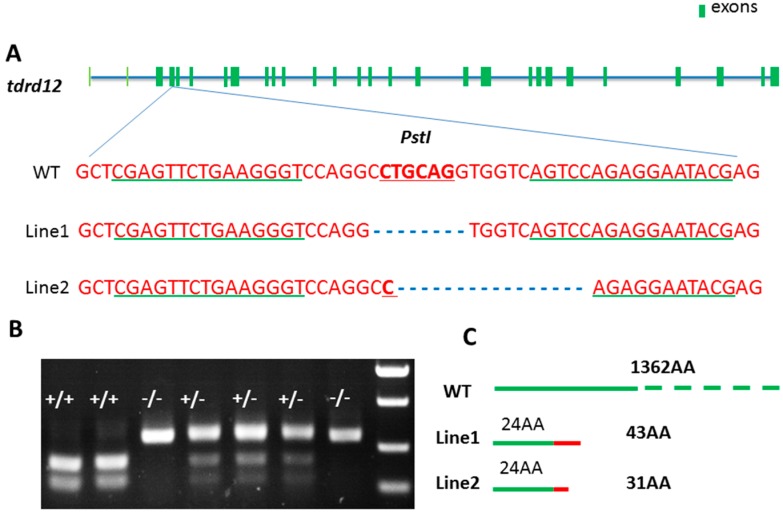
Generation of Tdrd12-deficient zebrafish by TALENs. (**A**) Binding site of engineered TALENS on the *tdrd12* gene exon 2 (exons shown in dark green). The underlined-green fonts indicate the sequences of the two targeting arms of TALENS, and the red fonts show the restriction enzyme *Pst1* cutting site. WT, wild-type. Mutant-line1 (M1) and mutant-line2 (M2) are two independent mutant lines in which the restrictive endonuclease site *Pst1* is eliminated with 8 and 16 bp deleted, respectively. Mutation confirmation as shown by the sequencing results of the transcripts of the *tdrd12* gene from the two mutant lines, M1 and M2; (**B**) Agarose gel electrophoresis image with the PCR products following *Pst1* digestion of the *tdrd12* locus of the F2 fish offspring. +/+, WT; +/−, heterozygous; and −/−, homozygous; and (**C**) Diagram representative of wild-type (WT) and two putative truncated mutant Tdrd12 proteins, M1 and M2, in which the first 24 amino acids (shown in green boxes) are identical to those of the wild-type Tdrd12 protein and the following 19 and 7 amino acids in M1 and M2, respectively (shown in red boxes), are miscoded ones. Both putative mutant forms of Tdrd12 are terminated prematurely.

To explore the functions and mechanisms of zebrafish Tdrd12, Tdrd12-deficient zebrafish were analyzed using TALEN in zebrafish. As the targeting region shown in Figure 3A, the 2nd exon of *tdrd12* was chosen as the target site, and the native *PstI* site was chosen as a screening tool for its loss in *tdrd12* mutants (Figure 3A,B). The primers used for *tdrd12* genotyping to amplify the target region for mutation screening are shown in Table 1. Two independent mutant lines were generated. The mutated *tdrd12* region fragments have been confirmed by sequencing, indicating that 8- and 16-bp deletion occurred in the mutant lines 1 and 2, respectively. These created two truncated Tdrd12 mutant forms in the mutant line 1 and mutant line 2 with only the first 24 amino acids of the N-terminus identical to native Tdrd12 in the wild-type fish (Figure 3C).

### 2.4. Tdrd12-Deficiency Results in Masculinization and Infertility in Zebrafish

**Figure 4:**
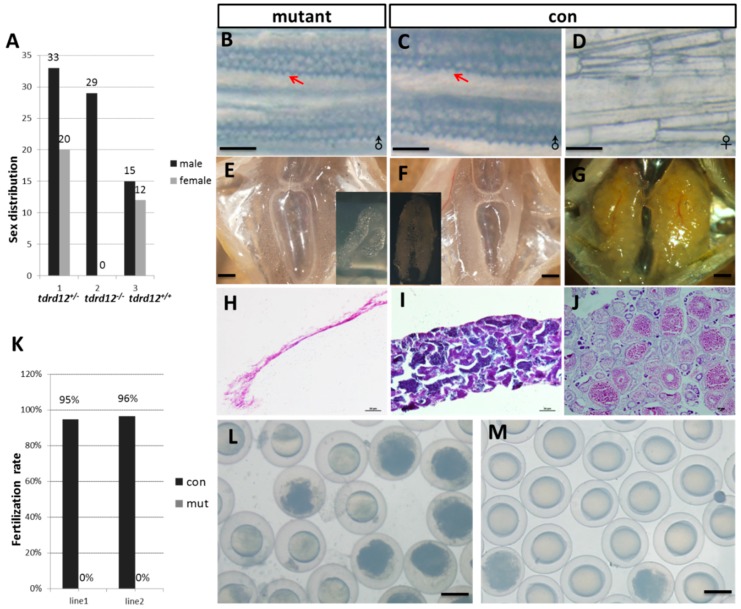
Tdrd12 deficiency results in masculinization and infertility in zebrafish. (**A**) Sex ratios of the progenies from the in-cross of *tdrd12* heterozygous mutant fish (90 dpf). Among the progenies, all homozygous offspring developed into males. The total number of progenies for this analysis is 109. *tdrd12*^+/−^: heterozygous progenies; *tdrd12*^+/+^: wild-type progenies; *tdrd12*^−/−^: homozygous progenies; (**B**–**D**) Appearance of the breeding tubercle (BT) clusters (red arrowheads) in the pectoral fin of all homozygous mutant fish (**B**) and wild-type males (**C**), but not in wild-type females (**D**), scale bar = 250 μm; (**E**–**G**) Anatomical views of the gonadal tissues of the Tdrd12-deficient fish and wild-type adults. Only atrophied testes were observed in Tdrd12-deficient adults (**E**), while normal testes (**F**) and ovary (**G**) could be observed in wild-types adults, scale bar = 500 μm; (**H**–**J**) Histological analyses of the gonadal tissues indicate no signs of germ cells in the atrophied testes of the Tdrd12-deficient adults (**H**), while normal spermatogenesis (**I**) and oogenesis (**J**) progresses in the wild-type adults, scale bar = 50 μm; (**K**) The fertilization rates of the mating between wild-type (*tdrd12^+/+^*) males and Tdrd12-deficient fish with wild-type females at the 120-dpf stage were recorded. The average fertilization rates of the 10 fish from three separate experiments were measured. Each group consists of 10 pairs of fish. The data shown here represent the means ± standard error of the mean; and (**L**,**M**) Morphological observations of embryonic development at 11 hpf derived from the mating between wild-type females with Tdrd12-deficient adults (**L**) and wild-type males (**M**); eggs from wild-type females could be induced by Tdrd12-deficient fish, but no successful fertilization could be found, scale bar = 250 μm.

All the Tdrd12-deficient fish were viable; however, no female could be found in *tdrd12*-null mutant adults (Figure 4A). To investigate whether the disruption of *tdrd12* would affect the formation of early primordial germ cells (PGCs) in zebrafish, whole-mount in situ hybridization of 2-dpf embryos from the in-cross of *tdrd12* heterozygous fish was performed using the *vasa* probe, the signals at the position of PGCs in the mutant were as normal as that in the wild-type or heterozygous fish (Figure S2). This result shows that the *tdrd12* mutant does not disrupt the formation of early PGCs. However, since maternal Tdrd12 in the Tdrd12-deficient embryos could be obtained from heterozygous parents, the conclusive function of Tdrd12 in the maintenance of PGCs could not be justified at the current stage.

At the adult stage (120-dpf), the mutants were all phenotypical males while the mixed male-female sex ratio in the wild-type siblings and heterozygous siblings was observed (Figure 4A). The identity of sex was based on the body pigmentation, shape of the abdomen, and epidermal tubercles (arrow heads in Figure 4B,C) on the pectoral fins, which specifically exist in the male characterization and filament-like testis (*n* = 12, Figure 4). When mated to females, the mutant males exhibited normal sex behavior and induced female egg laying. However, no successful fertilization of the eggs from wild-type female mating with the Tdrd12-deficient males was found (Figure 4K–M). These results reveal the loss of the germ cells in *tdrd12* mutant males.

**Figure 5:**
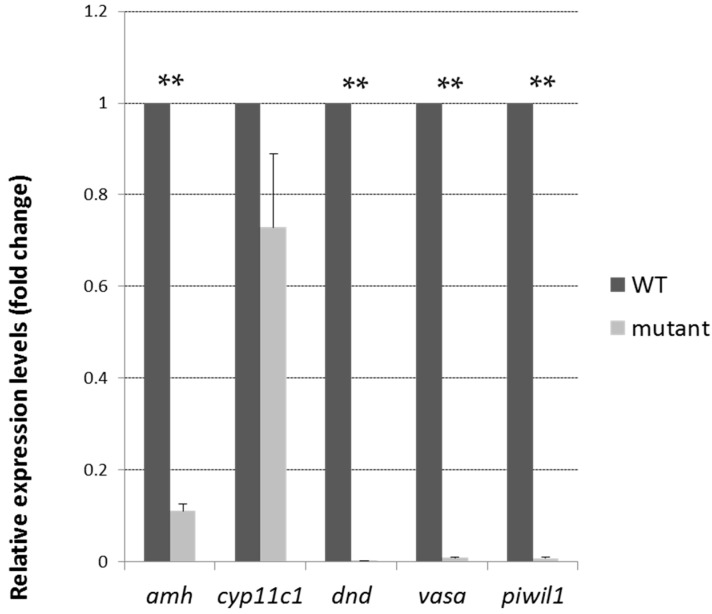
Existence of gonadal somatic cells, but not germ cells, in Tdrd12-deficient adults. Gene expression levels of several germ cell-specific genes (*vasa*, *dnd*, *piwil1*), a testis Sertoli cell-specific gene (*amh*), or a Leydig cell-specific gene (*cyp11c1*) in the filament-like testis of wild-type and Tdrd12-deficient adult testes samples were examined at the 90 dpf stage. ** *p* < 0.01 vs. wild type. *β-actin2* was selected as the most suitable and invariant reference gene for our samples from *gapdh*, *β-actin2*, and *ef1a* testing according to the published reports [33,34].

To investigate the mechanisms and reasons for the normal sex behavior but infertility in the mutant adult males, androgen concentrations in the blood and expression levels of germ cell-specific genes in the mutant and wild-type male siblings were measured at the adult stage. The expression levels of germ cell markers, such as *vasa*, *dnd*, the piRNA-Piwi-interacting gene *piwil1*, the male somatic cell marker *amh* (anti-Mullerian hormone), the earliest sex-specific male Sertoli cell marker and *cyp11c1* (cytochrome P450, family 11, subfamily C, polypeptide 1, also known as *cyp11b*), a male-specific Leydig cell marker, indicating androgen producing cells, were tested in the adult mutant testes and control testes (Figure 5). The results showed that the relative expression levels of the germ cell markers *vasa*, *dnd*, and *piwil1* were significantly reduced to undetected levels in the mutant testes. The expression of *amh* was reduced, but *cyp11c1* expression remained high in the mutants. The high expression of *cyp11c1* was somehow consistent with the high level of blood androgen (mutants: 374.3 pg/mL; wild-type: 234.75 pg/mL. *p* < 0.05) in the mutants, possibly indicating a compensatory response to the loss of the germ cells.

**Figure 6:**
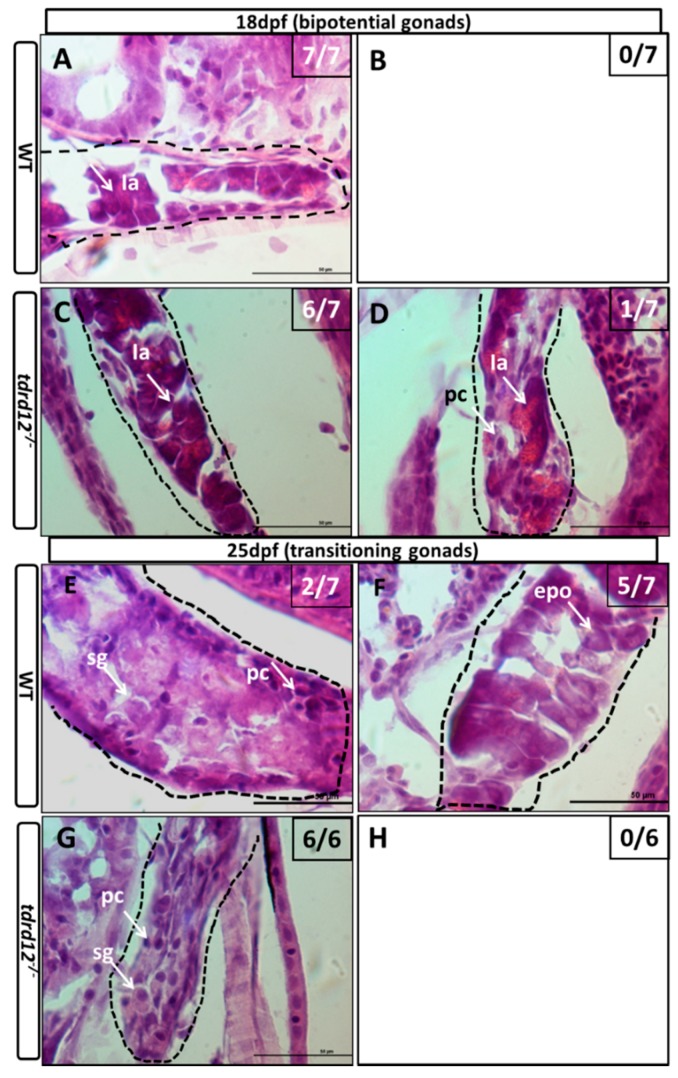

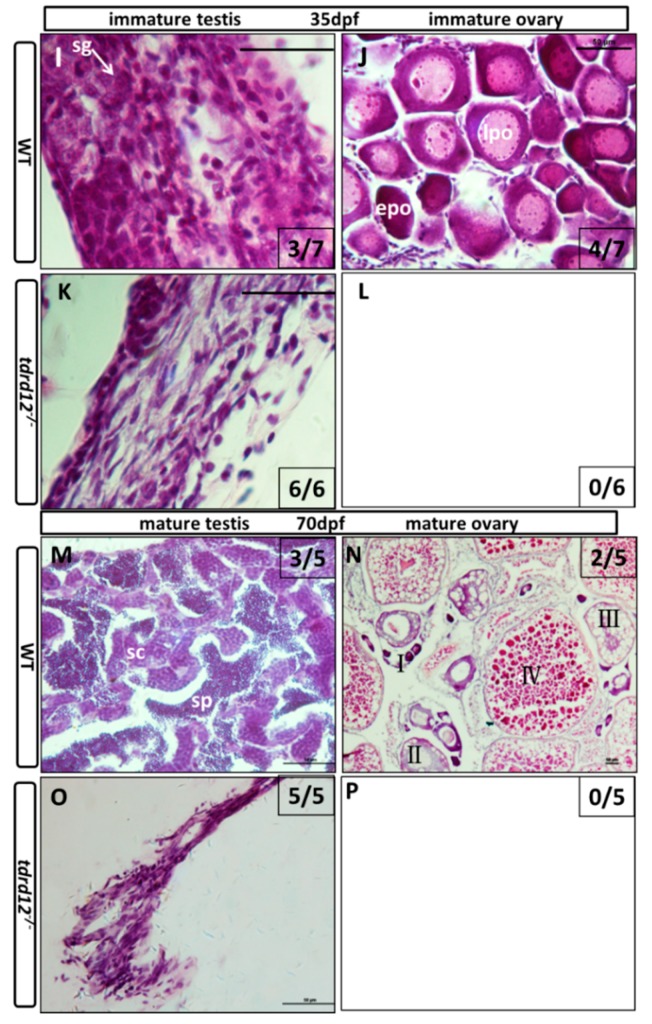
Masculinization occurred in Tdrd12-deficient zebrafish during the late “juvenile ovary” stage. Histological features with hematoxylin-eosin staining of cryo-sections were assessed from gonadal tissues in Tdrd12-deficient zebrafish and their wild-type siblings at various stages. (**A**–**D**) At the 18-dpf stage, typical stage Ia in the “juvenile ovary” was observed in all wild-type fish gonadal tissue (**A**, 7/7), and most Tdrd12-deficient fish gonadal tissue (**C**, 6/7). However, typical pyknotic cells (pc) in early testes could not been found in wild-type gonadal samples (**B**, 0/7) and only some of Tdrd12-deficient fish gonadal tissue samples (**D**, 1/7); (**E**–**H**) At the 25-dpf stage, typical pyknotic cells (pc) and spermatogonia (sg) in early testes were observed in some of the wild-type fish gonadal tissue (**E**, 2/7) and all Tdrd12-deficient fish gonadal tissues were examined (**G**, 6/6). However, the typical epo stage ((“early” perinucleolar oocytes): early stage IB) in early ovaries could be found in some of the wild-type gonadal tissue samples (**F**, 5/7). No ovary-like gonads could be found in Tdrd12-deficient gonadal samples (**H**); (**I**–**L**) At 35 dpf, typical spermatogonium (**I**) or oogonium (**J**) could be seen in the gonadal samples of the wild-type males (**I**, 3/7) and females (**J**, 4/7). However, only the filament-like testes with some Sertoli-like and Leydig-like cells, but no spermatogonium-like cells, were seen in the gonadal samples of the Tdrd12-deficient fish (**K**, 6/6). No ovary-like tissue was found in mutant fish (**L**, 0/6); (**M**–**P**). At 70 dpf, various spermatogonium (**I**) or oogonium (**J**) stages could be seen in the gonadal samples of the wild-type males (**M**, 3/5) and females (**N**, 2/5). However, only the filament-like testes without any sign of the presence of germ cells could be seen in all the gonadal samples of Tdrd12-deficient fish (**O**, 5/5). No ovary-like tissue has been found in mutant fish (**L**, 0/5). The gonadal tissues in (**A**–**H**) are circled with a black dotted line, and the white arrowheads indicate typical cell types in each pictures. (**A**–**H**,**I**,**K**): 1000×; (**J**,**M**,**O**): 400×; and (**N**): 100×. Scale bar for all pictures represents 50 μm. Pyknotic cells (pc), spermatogonia (sg), sperm (sp), spermatocytes (sc); stages of oogenesis: stage I, stage I is divided into stage Ia and Ib. (epo (“early” perinucleolar oocytes): early stage of Ib); lpo (“late” perinucleolar oocytes): late stage of Ib), **II**, **III**, and **IV**.

To follow the course of the gonad development of the Tdrd12-deficient fish from the heterozygous parents, the larvae of mutants and control siblings were first genotyped and then were sectioned at different developmental stages. At 18 dpf, undifferentiated gonads that contained early oocytes with no obvious morphological differences were observed in all of the wild-type sibling controls, and the *tdrd12* homozygous mutants (Figure 6A,C). The difference became accentuated between the *tdrd12* mutants and wild-type siblings at 25 dpf. All of the *tdrd12* mutant gonads lacked oocytes and had become testes with spermatogonia and pyknotic cells (sg and pc in Figure 6G); no ovary-like gonads were evident (Figure 6H). However, there were only two of seven wild-type siblings with a testis-like morphology that had numerous pyknotic cells and spermatogonia cells (pc and sg in Figure 6E) in the control siblings. The remaining control siblings had bipotential ovaries with stage IA and early stage IB (epo: “early” perinucleolar oocytes) oocytes according to Rodriguez-Mari et al. (Figure 6F) that prepare to progress through meiosis to enter the diplotene stage [6]. The morphological differences continued in another way at 35 dpf, with the shape of spermatogonia cells in the *tdrd12* mutant testes becoming deformed or apoptotic (Figure 6K). However, nearly half of the males had a large amount of spermatogonia cells in the immature testes, and the others were females with early oocytes in the immature ovaries (Figure 6I,J). At the stage of 70 dpf, consistent with the results observed at 35 dpf, all *tdrd12* mutants lacked oocytes and had tread-like testes without any germ cells but some somatic and Leydig cells (Figure 6O). By contrast, half of the wild-type controls had mature ovaries filled with oocytes at different stages of oogenesis (Figure 6N), and the other half had mature testes (Figure 6M) [6,32].

This histological analysis of larva at different developmental stages revealed that, in *tdrd12* mutant gonads, early oocytes that failed to progress to reach the diplotene stage at 18–35 dpf caused sex reversal in the sex-transitioning stage (Figure 6). Interestingly, the sex-reversal testes, compared with the wild type, showed abundant germ cell apoptosis or deformities (Figure 6K), resulting in thread-like infertile testes without any spermatozoa at the adult stage. These results suggest that the absence of oocytes, spermatozoa, and infertility could be related to the failure to complete meiosis associated with increased germ cell apoptosis.

The expression levels of some of the early gonad-specific somatic cell markers, such as *cyp19a1a* (*cytochrome P450 family 19 subfamily A polypeptide1a*, a female somatic cell marker specifically expressed in ovary granula cells), *amh*, and *cyp11c1* (male somatic cell markers) [35], were monitored with quantitative RT-PCR in *tdrd12* mutants, and their wild-type siblings at 13, 18, 25, and 35 dpf showed typical testicular endocrine features in Tdrd12-deficient adults (Figure S3).

### 2.5. Meiosis Defects of the Germ Cells in the tdrd12 Mutants in the Gonad-Transitioning Periods

**Figure 7:**
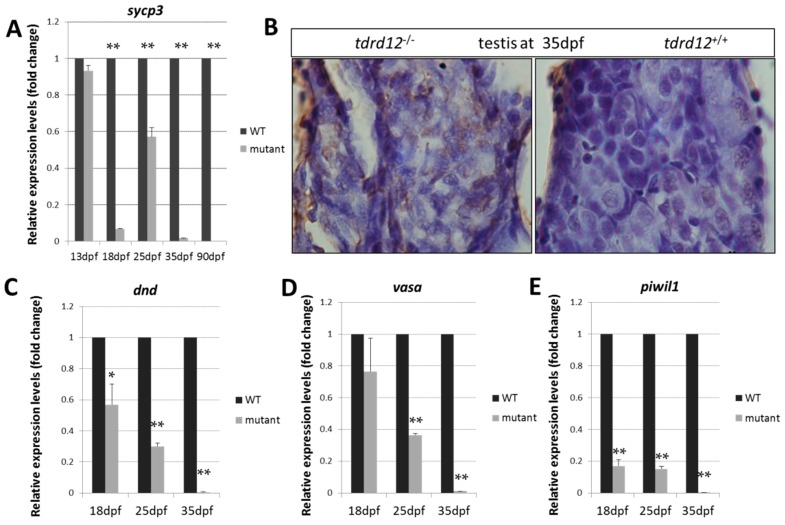
Meiotic defects and failure of germ cell maintenance in Tdrd12-deficient zebrafish. (**A**) Normalized expression of the meiotic recombination-specific gene *sycp3* (*synaptonemal complex protein 3*) in the developing gonads were examined in gonadal tissue samples in Tdrd12-deficient fish and their wild-type siblings at 13, 18, 25, and 35 dpf; (**B**) In situ cell death in the immature testis of *tdrd12^+/+^* and *tdrd12^−^^/^^−^* at 35 dpf, with abundant signals (**brown**) in the Tdrd12-deficient testis. Normalized expression of the germ cell-specific genes *dnd* (**C**), *vasa* (**D**), and *piwil1* (**E**) in the developing gonads were examined in gonadal tissue samples in Tdrd12-deficient fish and their wild-type siblings at 13, 18, 25, and 35 dpf. The tails of the larvae were collected for genotyping, while the rest of the body was used for total RNA isolation. The numbers of the examined fish for the assays at the 13, 18, 25, and 35 dpf stages for each genotype were 25, 20, 12, and 10, respectively. The experiments were performed with three biological repeats. * indicates the difference at *p* < 0.05 vs. wild type; ** indicates the significant difference at *p* < 0.01 vs. wild type. *β-actin2* was selected as the most suitable and invariant reference gene for our samples from *gapdh*, *β-actin2*, and *ef1a* testing according to the published reports [33,34].

The early meiotic marker *sycp3* (Synaptonemal complex protein 3), which is required to assemble the synaptonemal complex during meiotic prophase was used to detect the meiosis defects in the germ cells at 18, 25, 35 dpf, and the adult stage. The expression levels of *sycp3* were nearly undetectable in the mutants at the critical time of the meiosis period at 18, 25 dpf, and the adult stages (Figure 7A), and abundant apoptosis was seen in the Tdrd12-deficient testis (Figure 7B). These results were consistent with the previous histological results of the meiosis defects and germ cell death in the mutants.

The expression levels of germ cell markers *dnd*, *vasa*, and *piwil1* in the gonad-transitioning periods at 18, 25, and 35 dpf were examined (Figure 7C–E). The results showed that the expression levels of the germ cell-specific genes in the mutants were slightly down-regulated compared with those in the wild-type siblings at 18 dpf. However, these expression levels were significantly reduced by approximately three-fold relative to those in the wild-type siblings at 25 dpf. Finally, dramatically reduced expression levels of these germ cell-specific genes were observed in *tdrd12* mutants when the gonads were at the developmental stage of 35 dpf (Figure 7C–E). The loss of the germ cells somehow resulted from the meiotic defects and reflect the previous histological defects observed at these developmental stages.

## 3. Discussion

In mammals, 12 members of the TDRD protein family have been identified since *Drosophila* Tudor proteins were discovered [18,20,22,23,24,29,30,36,37,38]. We also demonstrated that our newly identified Tdrd12 is most homologous to mouse and human TDRD12 among the 12 TDRD family members. The orthologs of these 12 members of Tdrd family proteins, which were all Tudor-domain-containing proteins are also shown in Table 2 in zebrafish. Previous studies have shown that many family members of Tudor-domain-containing proteins are involved in germ cell development in mice. In mice, mutants lacking the Tudor domain-containing proteins such as TDRD1, TDRD5, TDRD6, TDRD9, and TDRD12 resulted in male sterility, and their functions and interactions with the PIWI protein are becoming increasingly studied [21,23,29,39,40,41]. It was reported in mice that TDRD12 was detected in the cytoplasm of primary spermatocytes and acrosomes of the spermatid at 4 weeks old when the round spermatids were present in the seminiferous tubule [30]. The disruption of *tdrd12* in mice caused male infertility and is dispensable for primary piRNA biogenesis, but is essential for the production of secondary piRNAs. Despite its extensive conservation across species, the functions and mechanisms of TDRD12 in germ cells were similar between mice and flies in that it was required for germ cell development and reproduction, although it is required for spermatogenesis in mice and for oogenesis in *Drosophila* [18,29].

In this study, we provided the first description of *tdrd12* and generated two homozygous mutants to show that maternally provided Tdrd12 is likely to fulfill any potential roles for early germ cell migration, proliferation, or maintenance at the early development stages (Figure S1), even for the initial formation at the ovary-like bipotential stages. However, at the sex-transitioning stages, our findings indicate that zygotic *tdrd12* is required for the morphology and maintenance of germ cells. We also note that germ cells in the *tdrd12* mutants have a peculiar karyopyknosis appearance at the pachytene stages of 25 and 35 dpf, a possible indication of germ cell death. The phenotype of sterile males of *tdrd12* mutants is reminiscent of the phenotypes displayed in genes required for germ cell survival and maintenance in zebrafish, including *ziwi* and *zili*, and DNA-damage repair genes *brca2*, *fancl*, *vasa*, and *tdrd1* [6,9,15,16,26,42]. Although little is known about how these analogous proteins interact with PIWI proteins in zebrafish, a similar phenotype in Tdrd12-deficient fish with the mutants of the piRNA-processing component *ziwi* or *zili* of zebrafish with increased germ-cell apoptosis during the establishment and differentiation of the juvenile gonad have been observed [6,15,16,42]. It has been observed that the sterile male phenotype of our *tdrd12* mutants also resulted from the lack of germ cells due to their meiosis defects. Our anatomical and histological morphology analyses, along with the examination of the expression levels of specific germ cell markers such as *vasa*, *dnd*, and *piwil1*, and meiotic marker *sycp3* indicated the strong defects of the maintenance and meiosis in germ cells caused by the Tdrd12 deficiency. In contrast to wild-type males, the gonad in the mutants forms a filament-like testis without germ cells resulting from the meiosis defects. These mutants containing somatic and Leydig cells could produce androgens normally compared with the wild-type sibling testes. All of the results showed the absolute loss of the germ cells caused by the meiosis defects, while the normal male somatic fate remained.

Zebrafish Tdrd12 possesses two Tudor domains and a dead box, such as many other Tudor proteins (Table 2). These conserved domains may play a critical role in protein-protein interactions. However, the precise functions and mechanisms of these domains differ among the reported Tdrd1, Tdrd6, and Tdrd9 in zebrafish [26,28]. The *tdrd1* mutants and most of the *tdrd9* mutants were sterile, resulting from the loss of germ cells. However, some *tdrd9* mutant individuals maintain germ cells until adulthood and can produce functional gametes at low fecundity. Progenies resulting from these *tdrd9* mutant gametes develop normally, indicating that no major defects occurred during the development of these mutant gametes [28]. Although *tdrd6* is also required for normal primordial germ cell formation and the accumulation of maternally inherited piRNAs, the mutant males were fertile [28]. This is opposite to our findings that *tdrd12* mutants begin with normal development and numbers of early germ cells at the onset of gametogenesis, but the meiosis of the germ cells is disrupted, and the mutants become sterile with the germ cells completely depleted. The loss of germ cells is very similar to that occurring in *piwi* and *tdrd1* mutants in zebrafish [15,16,26] and to the Tdrd12 ortholog in *Drosophila* that is known to interact with the essential piRNA pathway and regulate piRNA biogenesis in ovarian germ line cells [15]. TDRD12 in mice was also identified as a component of the PIWI protein MIWI2. TDRD12 deficiency in male mice induces male testes atrophy resulting from the loss of MIWI2-bound piRNA that is important for secondary piRNA biogenesis [29]. It was reported that *vasa* is also integral to the piRNA biogenesis pathway, acting as a scaffold for the transposon transcripts processed by Piwi proteins in the piRNA ping-pong amplification loop [9,43]. These molecular factors may indicate that Tdrd12 acts in the Piwi-piRNA pathway in zebrafish. Unfortunately, in the absence of functional antibodies and rescuing transgenes, we are unable to further probe the subcellular localization of Tdrd12 and show the interactions between Tdrd12 and Piwi proteins or other Tdrd proteins. The *tdrd12* mutant phenotype displays the same or severe phenotype as either the *zili* or *ziwi* mutant. In the *tdrd12* mutant, germ cells do develop and differentiate, although they have a meiotic defect and cannot be maintained; however, in *piwi* mutant animals, the germ cells do not reach these stages [15,16]. The relative normal progress regarding the early PGC migration and development of undifferentiated gonads has been observed in our Tdrd12-deficient fish, possibly because of the partial maternal rescue of Tdrd12 in the mutant embryos from their heterozygous parents. In the absence of Tdrd12 antibodies, however, this cannot be tested. However, the phenotype triggered by the loss of *tdrd12* in the adult stage may simply be stronger than that described previously for *tdrd6* and *tdrd9* [28].

In zebrafish, sex determination is complex, and environmental factors such as the water temperature or growth rate can influence the sex ratio [44]. The complete loss of early PGCs caused by the depletion of Dnd promotes masculinization and sterility [10]. Masculinization of the gonad was also affected by the germ-cell numbers when it falls below a threshold [11,12,13,45]. In this work, the maternal and germ cell-related expression pattern is observed, which is similar to some of the other germ cell markers: *vasa* and *dnd* [10,46]. The maternally provided mRNA gradually vanished, and the zygotic expression begins at approximately 15 dpf and was continually expressed and established throughout the sex-transitioning stages until the adult gonads of both sexes. Although we did not observe obvious defects in the PGCs and formation of the “bipotential juvenile ovaries” in our Tdrd12-deficient progenies from the *tdrd12* heterozygous parents, which could be the partial rescue effects due to the inheritance of the maternal Tdrd12 presence in the Tdrd12-deficient embryos, the failure to maintain the essential progress of the meiosis of the germ cells and gametogenesis was evident in Tdrd12-deficient fish at later stages. Along with the decreased numbers and eventual loss of all the germ cells, masculinization of the gonads and sterility have been observed as the defects caused by the germ cell-free phenotype reported previously in *dnd*-morphants [10]. This again further suggested the essential roles of Tdrd12 in the maintenance and development of zebrafish germ cells, and the conception of the critical signals derived from sufficient germ cells for the female fate of fish gonads.

Tdrd protein families have been rarely reported thus far, and their functions and mechanisms remain unknown in zebrafish. Tdrd1 was reported to associate with piRNA targets interacting with both Ziwi and Zili in zebrafish. The loss of Tdrd1 leads to less electron density or the absence of nuage structures in germ cells, Piwi pathway-related defects such as germ-cell defects and transposon desilencing, which were consistent with the results obtained in mice [26,27]. Tdrd9 is also required for germ cell maintenance and affects piRNA accumulation, resulting in strong germ cell developmental defects in mutant zebrafish [28]. Tdrd6 is required for normal primordial germ cell formation and the accumulation of maternally inherited piRNAs in zebrafish. However, the *tdrd6* mutants show normal germ cell development in the adults [28]. Our current work identified the first teleost Tdrd12 containing two Tudor domains and a DEAD box in zebrafish. It has been demonstrated that the Tdrd12 ortholog in zebrafish is essential for the progress of the meiosis of germ cells and gametogenesis. Tdrd12 deficiency in zebrafish resulted in masculinization and sterility, which might be due to the loss of MIWI2-bound piRNA, which is important for secondary piRNA biogenesis [29].

## 4. Materials and Methods

### 4.1. Animal Maintenance

Our zebrafish (domesticated AB strains) were raised in a circulating water system at 28.5 °C according to standard methods. Embryos were obtained by natural spawning and were cultured in egg water. The culture of zebrafish and all experimental procedures were conducted according to the National Guide for the Care and Use of Laboratory Animals and were approved by the Ethics Committee from the Institute of Hydrobiology, Chinese Academy of Sciences (Approval No. IHB20110089).

### 4.2. tdrd12 Knock out by TALENs

Paired TALENs were designed and assembled as described previously [47] using the Golden Gate TALEN Kit (Addgene, Cambridge, MA, USA). The DNA sequences that were targeted by each TALEN pair are shown in Figure 3A. A native enzyme restriction site, *PstI*, in the spacer region between the two TALEN arms was used for nucleotide change detection. Next, mRNA was synthesized using the T3 mMESSAGE mMACHINE Kit (Ambion, Austin, TX, USA) using the backbone plasmids pCS2TAL3DD and pCS2TAL3RR. Approximately 200–500 pg of mRNA was microinjected into 1- to 2-cell-stage zebrafish embryos. Next, 10–20 embryos following their hatching were collected for genotyping, and the target region was amplified using the primers in Table 1. In this assay, the remainder of the embryos was raised to adults and were mated with wild-type zebrafish to generate F1 offspring. The F1 adults were genotyped by enzyme digestion and PCR product sequencing. The F2 generation was obtained from F1 strains harboring the mutations.

### 4.3. RT-PCR and Quantitative Real Time PCR (Q-PCR)

Total RNA was purified from different stages of embryos, juveniles, and various adult tissues following the standard protocols using the Trizol reagent (Invitrogen, Carlsbad, CA, USA). The cDNAs were synthesized using the Reverse Aid First-Strand cDNA Synthesis Kit (K1622, Thermo Scientific, Waltman, MA, USA) according to the manufacturer’s instructions. Quantitative real-time PCR primers were designed using “primer blast” in the National Center for Biotechnology Information (NCBI) database. In the Q-PCR assays, *β-actin2* was selected as the most suitable and invariant reference gene for our samples from *gapdh*, *β-actin2*, *ef1a* testing, according to the published reports [33,34]. Q-PCR was performed using Bio-Rad IQ SYBR Green SuperMix. Each experiment was performed in biological and sample triplicates. The relative mRNA levels were normalized to *β-actin2* and calculated by the normalized CT values (2^−ΔΔ*C*t^), compared with wide-types at each time point. The results were expressed according to a previously described method [48]. The transcript of zebrafish *β-actin*2 was amplified as the internal control for RT-PCR. All primers for tdrd12 and β-actin2 used in the RT-PCR and primers used in Q-PCR assay are summarized in Table 1.

### 4.4. Genomic DNA Isolation and Genotyping

Whole embryos, as well as juvenile and adult caudal fins, were cut for genomic DNA isolation according to NaOH assays, as previously described [49]. The DNA was then used for genotyping.

### 4.5. Masculinization and Fecundability Assessments

The sex ratio was calculated after maturation at 3 mpf (month post fertilization), and BT cells, which exist only in male pectoral fins, were detected using microscopy (Olympus Corporation, Tokyo, Japan). The mutant males and their wild-type male siblings from 2 independent lines were mated with wild-type females for three valid mating trials independently, with each group consisting of 10 fish. The average fertilization rate for each mating was recorded based on the proportion of fertilized eggs to total spawning eggs. Males were individually bred during each trial.

### 4.6. Androgen Measurement

The androgen concentrations in the blood of the mutant and wild-type male fish were measured at 3 months post fertilization (mpf) according to commercial ELISA kits (Cayman Chemical Company, 582701 and 582751, Ann Arbor, MI, USA). First, the adult fish were anesthetized; blood was collected from the caudal vein (blood from 3 individual fish was pooled as one sample) and then was centrifuged at 1000× *g* for 5 min at 4 °C. Approximately 10 μL of the supernatant was harvested and diluted to 400 μL with double distilled water. After extraction twice with 2 mL of diethyl ether, the supernatant was collected and evaporated with nitrogen. Finally, the residue was dissolved with 120 μL of EIA (enzyme immunoassay) buffer and then was measured following the manufacturer’s instructions. There were three replicate samples for the *tdrd12* mutant males and wild-type male siblings.

### 4.7. Anatomical and Histological Assays

For the preparation of tissue sections and RNA isolation, the juveniles or adults were first euthanized with 100 mg/L tricaine methanesulfonate (Ardrich, St. Louis, MO, USA), and various tissue samples or larva trunks were collected. Tissue samples for RNA isolation were stored in Trizol for RNA isolation; the samples for section-staining assays were fixed in 4% PFA (paraformaldehyde) for at least 2 days, washed in PBS, and balanced in 30% sucrose for at least 24 hpf until the tissues fall down to the button. Next, the tissues were embedded in OTC overnight and cryo-sectioned. H&E (hematoxylin and eosin) staining was performed according to the standard procedures.

### 4.8. In Situ Hybridization Assays

For whole-mount in situ hybridization, the embryos from the in-cross of *tdrd12* heterozygous fish at 2 hpf were collected and fixed in 4% PFA overnight at 4 °C and then were dehydrated in pure ethanol at −20 °C when needed. Next, the embryos were hybridized with the *vasa* probe following standard protocols. The embryos from gonadal tissues of wild type were obtained from 4-month-old fish. The embryos and cryo-sectioned slides were then hybridized with the *tdrd12* probe according to the standard protocols.

### 4.9. In Situ Cell Death

The larval gonads of *tdrd12^−^^/^^−^* and *tdrd12^+/+^* were dissected and paraffin-sectioned at 6-µm thickness. The TUNEL (Terminal-deoxynucleoitidyl Transferase Mediated Nick End Labeling) assay was performed according to the instructions of the In Situ Cell Death Detection Kit, peroxidase (POD) (Roche Diagnostics Gmbh, Mannheim, Germany). The sections were then stained with hematoxylin. Images were recorded using a microscope (Leica microsystems, Wetzlar, Germany).

### 4.10. Fecundity Assessment

Adult Tdrd12-deficienct males and their control sibling males were crossed with wild-type females for three different valid mating trials every week, and the average fertilization rates of the 10 fish were measured, the fertilization rate was calculated as the ratio of fertilized eggs to the total eggs spawned. The data shown here represent the means ± standard error of the mean.

### 4.11. Statistical Analysis

The fecundability and quantitative real-time data were analyzed by non-parametric tests and by Student’s *t*-test using SPSS (SPSS22.0, Inc., Chicago, IL, USA). *p* ≤ 0.01 was considered statistically significant.

## 5. Conclusions

In this study, zebrafish *tdrd12* mRNA sequence was identified. The phylogenetic analyses of the predicted amino acid sequence of this zebrafish Tdrd12 with the other Tdrd12 reveal highly evolutionary and phylogenetic relationships among species. The maternal and germ cell-specific expression patterns of zebrafish *tdrd12* were confirmed subsequently. Utilizing TALEN (transcription activator-like effector nuclease) techniques, two independent *tdrd12* mutant zebrafish lines were generated. Although no defects of the generation and migration of the PGCs were observed. All Tdrd12-deficient mutants develop as infertile males by 35-dpf exclusively. This indicates the requirement of zygotic Tdrd12 for germ cell development and maintenance at the zebrafish juvenile stage. Our data indicate that the failure to support germ-cell development in Tdrd12-deficient fish is due to the meiosis defects that progress beyond the pachytene stage and the loss of germ-line stem cells eventually, both of which cause impaired testes without any germ cells. Thus, zebrafish Tdrd12 appears to be required for the development and maintenance of germ cells. We undertook a comprehensive analysis of the function of Tdrd12 in zebrafish, the results of which may shed light on its critical role in the piRNA pathway during gametogenesis.

## Figures and Tables

**Table 1:** Primers used in the experiments.

Gene	Forward primer (5′–3′)	Reverse primer (5′–3′)	Product Length
*tdrd12*-RT	GAGGAAACGGAGCACGTGTA	GGCTGAACTCTGCACAGGAT	941 bp
*tdrd12*-mRNA	CGCGCGATTTTAGAAATTGGAG	TGTTCAGTCGCTGTCGCTGTCGCT	4111 bp
*tdrd12*-genotype	GGTTTCAGTGTGATCATGGCTC	TAAACCGCTGCATTACGCACT	296 bp
*tdrd12*-probe	TGTCGAGCTGTGGTTGAGTC	TAATACGACTCACTATAGGGACT (T7)TGTCATCGGTTTGACCAGGG	841 bp
*cyp11c1-*real time	AACCCTGATGTGCAGGAGTG	TGAACGGTGATTCCCACAGG	152 bp
*amh*-real time	TGACTGAACTGAGTGCGCTT	CATCTGCAGGGCTTTCAGGA	110 bp
*cyp19a1a*-real time	TGCACAGATCCGAATTCTTCT	GCTGCGACAGGTTGTTGGTTT	237 bp
*ziwi (piwil1)*-real time	TGACATAACAGATGGCAACCA	GCCCTCTCTCTGTTCAGGACT	202 bp
*dnd*-real time	TGATTCCTCAACCCACCATAA	TGGACTTCATATTGCGGAGA	202 bp
*vasa*-real time	GGGCTGCAATGTTCTGTGTG	CAGTTTGCGCATTTCTGGCT	148 bp
*sycp3*-real time	GGCAGAAGCTGACCCAAGAT	TTTTGCACAACCCTTGCCTG	154 bp
*β-actin2*-real time	GATGATGAAATTGCCGCACTG	ACCAACCATGACACCCTGATGT	135 bp

**Table 2:** Identification and classification of Tdrd family members in zebrafish.

Tudor-Containing Proteins	Chr Location/Protein	Mouse Orth.	Domains	Functions in Zebrafish	References
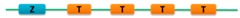	12/Tdrd1 (1176 AA)	TDRD1	4×Tudor, 1×zf-MYND	Germline development	[26,28]
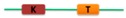	12/Tdrd2/Tdrkh (573 AA)	TDRD2/TDRKH	1×Tudor, 1×KHI	Not reported	
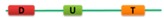	11/Tdrd3 (905 AA)	TDRD3	1×Tudor, 1×DUF, 1×UBA	Not reported	
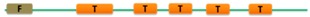	9/Tdrd4/Rnf17 (1521 AA)	TDRD4/RNF17	5×Tudor, 1×FYVE	Not reported	
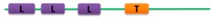	22/Tdrd5 (905 AA)	TDRD5	1×Tudor, 3×LOTUS	Not reported	
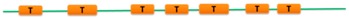	20/Tdrd6 (2177 AA)	TDRD6	7×Tudor	Germ plasm assembly	[28]
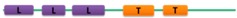	1/Tdrd7 (1079 AA)	TDRD7	2×Tudor, 3×LOTUS	Granule number and morphology	[31]
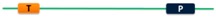	16/Tdrd8/Stk31 (977 AA)	TDRD8/STK31	1×Tudor, 1×PKc-like	Not reported	
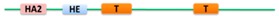	13/Tdrd9 (1342 AA)	TDRD9	2×Tudor, 1×HA2, 1×HELICC	Germline development	[28]
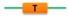	17/Tdrd10/Smndc1 (237 AA)	TDRD10/SMNDC1	1×Tudor	Not reported	
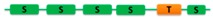	4/Tdrd11/Snd1 (913 AA)	TDRD11/SND1	1×Tudor, 5×SNc	Not reported	
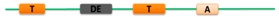	25/Tdrd12 (1362 AA)	TDRD12	2×Tudor, 1×DEXDc, 1×ACD	This paper	

Cartoon showing all zebrafish proteins containing tudor domains (orange boxes). All significant protein domains identified via HHpred searches or the NCBI database are indicated with colored boxes. The domains given above (HE stands for HELICc: helicase superfamily c-terminal domain; D stands for DUF: domain of unknown function; U stands for UBA: ubiquitin-associated domain; Z stands for zf-MYND: MYND finger; T stands for TUDOR: Tudor domain; L stands for LOTUS: an uncharacterized small globular domain found in Tudor-containing proteins 5 and 7; HA2: helicase-associated domain; SNc: Staphylococcal nuclease homologs; K stands for KHI: K homology RNA-binding domain, type I; F stands for FYVE domain: Zinc-binding domain; P stands for PKc-like: protein kinases; A stands for ACD: α-crystallin domain; D stands for DEXDc: DEAD-like helicase superfamily). The number of identified TUDOR and other domains in zebrafish and their orthologs found in the mouse is shown to the right. The functions of *tdrd1*, *tdrd6*, *tdrd7*, and *tdrd9* in zebrafish were previously reported and the relevant publications are noted in Table 2.

## Supplementary Materials

Supplementary materials can be found at www.mdpi.com/1422-0067/18/6/1127/s1.
