# Supplementary material for: Tdrd12 Is Essential for Germ Cell Development and Maintenance in Zebrafish
Source: Int J Mol Sci. 2017 Jun 7;18(6):1127. doi: 10.3390/ijms18061127 (PMC5485951; doi:10.3390/ijms18061127)
Supplement: Supplementary file 1 [file ijms-18-01127-s001.pdf]

Supplementary Materials:

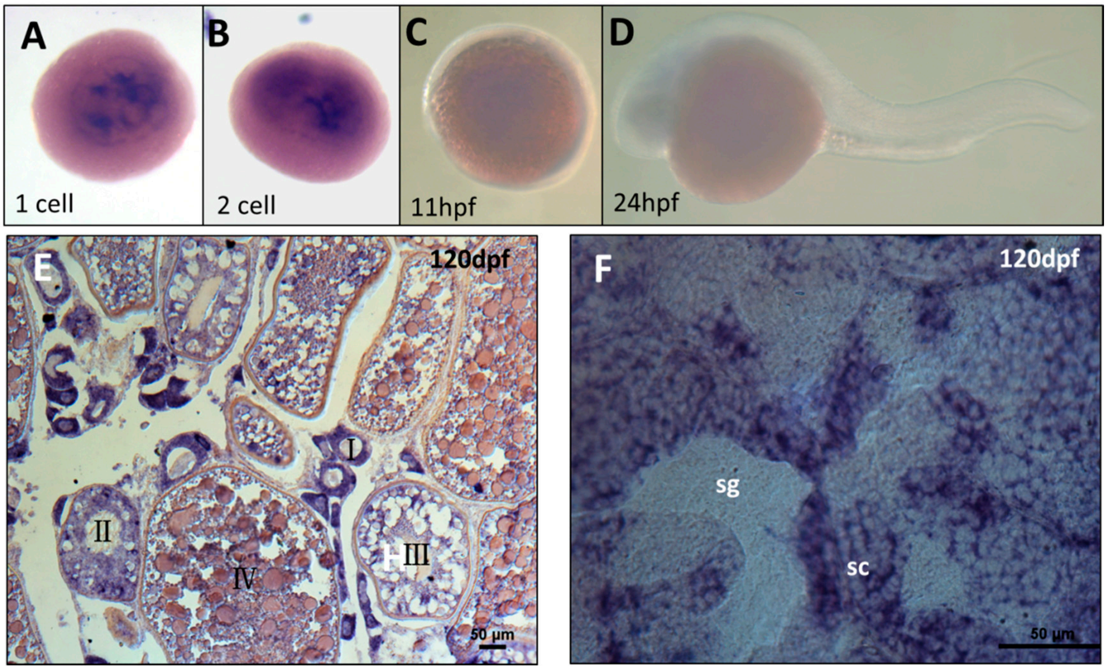

Figure S1. Expression pattern of *tdrd12* in the germ cells. In situ hybridization of the *tdrd12* probe in embryos at the 1- to 2-cell stage (**A** and **B**), 11 hpf (**C**), 24 hpf (**D**), and cryo-sections of adult gonads are present in the ovary (**E**) and testis (**F**). The types and stages of the cells were labeled, and the signals are indicated in purple. However, the signals vanished at 11 hpf and 24 hpf (**C** and **D**).

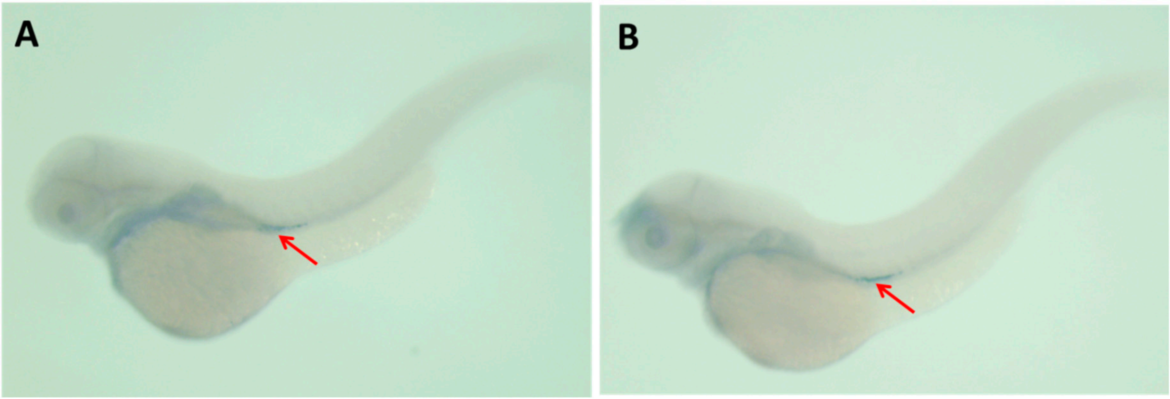

Figure S2. Normal development of the PGCs seen in *tdrd12*-deficient larvae from heterozygous parents. (**A**,**B**) PGCs visualized with the *vasa* riboprobe in wild-type larvae (**A**) and *tdrd12*-deficient larvae from the heterozygous parents (**B**) at 24 hpf. PGCs are indicated with arrowheads.

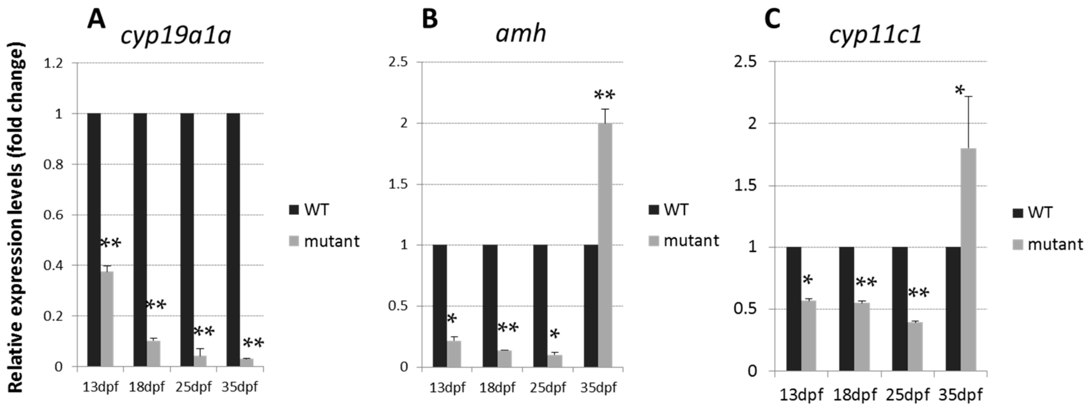

Figure S3. Typical testicular endocrine features remain in *tdrd12*-deficient adults. (**A, B, C**) Normalized expression of the female somatic cell marker *cyp19a1a* (**A**), male somatic cell marker *amh* (B), and Leydig cell marker *cyp11c1* (**C**) were examined in gonadal tissue samples in *tdrd12*-deficient fish and their wild-type siblings at 13 dpf, 18 dpf, 25 dpf, and 35 dpf. The tails of the larvae were collected for genotyping, while the rest of the body was used for total RNA isolation. The numbers of the examined fish for the assays at the 13-dpf, 18-dpf, 25-dpf, and 35-dpf stages for each genotype were 25, 20, 12, and 10, respectively. *β-actin2* was used as the reference gene for our samples. The experiments were performed for 3 biological repeats. * indicates the difference at $p < 0.05$ vs. wild type; ** indicates the significant difference at $p < 0.01$ vs. wild type.
